# Thermal limits in native and alien freshwater peracarid Crustacea: The role of habitat use and oxygen limitation

**DOI:** 10.1111/1365-2435.13050

**Published:** 2018-02-06

**Authors:** Wilco C. E. P. Verberk, Rob S. E. W. Leuven, Gerard van der Velde, Friederike Gabel

**Affiliations:** ^1^ Department of Animal Ecology and Physiology Institute for Water and Wetland Research (IWWR) Radboud University Nijmegen The Netherlands; ^2^ Department of Environmental Science Institute for Water and Wetland Research (IWWR) Radboud University Nijmegen The Netherlands; ^3^ Netherlands Centre of Expertise on Exotic Species (NEC‐E) Nijmegen The Netherlands; ^4^ Naturalis Biodiversity Center Leiden The Netherlands; ^5^ Institute of Landscape Ecology University of Münster Münster Germany

**Keywords:** amphipods, global warming, hypoxia, invasive species, isopods, pollution

## Abstract

1. In order to predict which species can successfully cope with global warming and how other environmental stressors modulate their vulnerability to climate‐related environmental factors, an understanding of the ecophysiology underpinning thermal limits is essential for both conservation biology and invasion biology.
2. Heat tolerance and the extent to which heat tolerance differed with oxygen availability were examined for four native and four alien freshwater peracarid crustacean species, with differences in habitat use across species. Three hypotheses were tested: (1) Heat and lack of oxygen synergistically reduce survival of species; (2) patterns in heat tolerance and the modulation thereof by oxygen differ between alien and native species and between species with different habitat use; (3) small animals can better tolerate heat than large animals, and this difference is more pronounced under hypoxia.
3. To assess heat tolerances under different oxygen levels, animal survival was monitored in experimental chambers in which the water temperature was ramped up (0.25°C min^−1^). Heat tolerance (CTmax) was scored as the cessation of all pleopod movement, and heating trials were performed under hypoxia (5 kPa oxygen), normoxia (20 kPa) and hyperoxia (60 kPa).
4. Heat tolerance differed across species as did the extent by which heat tolerance was affected by oxygen conditions. Heat‐tolerant species, for example, *Asellus aquaticus* and *Crangonyx pseudogracilis*, showed little response to oxygen conditions in their CTmax, whereas the CTmax of heat‐sensitive species, for example, *Dikerogammaru*s *villosus* and *Gammarus fossarum*, was more plastic, being increased by hyperoxia and reduced by hypoxia.
5. In contrast to other studies on crustaceans, alien species were not more heat‐tolerant than native species. Instead, differences in heat tolerance were best explained by habitat use, with species from standing waters being heat tolerant and species from running waters being heat sensitive. In addition, larger animals displayed lower critical maximum temperature, but only under hypoxia. An analysis of data available in the literature on metabolic responses of the study species to temperature and oxygen conditions suggests that oxygen conformers and species whose oxygen demand rapidly increases with temperature (low activation energy) may be more heat sensitive.
6. The alien species *D. villosus* appeared most susceptible to hypoxia and heat stress. This may explain why this species is very successful in colonizing new areas in littoral zones with rocky substrate which are well aerated due to continuous wave action generated by passing ships or prevailing winds. This species is less capable of spreading to other waters which are poorly oxygenated and where *C. pseudogracilis* is the more likely dominant alien species.

A http://onlinelibrary.wiley.com/doi/10.1111/1365-2435.13050/suppinfo is available for this article.

## 1 INTRODUCTION

Freshwater animals face new thermal challenges owing to climate change, local discharge of cooling water and to globalization resulting in redistribution and invasions of species into habitats with a different thermal regime. Patterns in the geographical distribution of aquatic ectotherms have been linked to their thermal tolerance and hypoxia tolerance (e.g. Calosi, Bilton, Spicer, Votier, & Atfield, 2010; Deutsch, Ferrel, Seibel, Pörtner, & Huey, 2015). Increasing our understanding of the physiological mechanisms underlying thermal responses is essential for both conservation biology and invasion biology as it enables us to better predict which species can successfully cope with global warming and how other environmental stressors modulate their responses to changing thermal regimes of their habitat (Chown, 2012; Huey et al., 2012; Verberk & Bilton, 2013). With increasing temperature, the demand for oxygen increases more than the rate at which oxygen can be supplied, due to capacity limitations (Fry & Hart, 1948; Pörtner, 2010; Verberk, Bilton, Calosi, & Spicer, 2011; Winterstein, 1905). Consequently, oxygen could become limiting, constraining aerobic energy metabolism necessary for reproduction, growth, and physical activities, including predator avoidance, feeding, and locomotion (Pörtner, 2010). However, there is an ongoing debate on the universality of this oxygen‐ and capacity‐limited thermal tolerance (OCLTT) hypothesis and the validity of its assumptions (e.g. Ern et al., 2015; Jutfelt et al., 2014; Klok, Sinclair, & Chown, 2004; Pörtner, 2014; Verberk, Overgaard, et al., 2016).

The problem of insufficient oxygen under warm conditions may be more immediate in water than in air as aquatic gas exchange is challenging due to the lower rate of oxygen diffusion in water and the larger effort required for ventilation as water has a higher density and viscosity (Verberk & Atkinson, 2013). Similarly, this challenge of breathing underwater is thought to explain the recurrent evolution of air breathing in crustaceans from tropical waters that may be more prone to severe hypoxia (Fusi et al., 2016; Giomi et al., 2014). Moreover, oxygen availability is more variable in an aquatic setting, declining at night and increasing during the day by primary producers, and options for thermoregulation to adaptively modulate body temperature are limited. Consequently, interactions between warming, dissolved oxygen concentrations and organic pollution may be relevant especially in aquatic systems (Diaz & Rosenberg, 2008; Meire, Soetaert, & Meysman, 2013; Moran & Woods, 2010; Posch, Köster, Salcher, & Pernthaler, 2012; Verberk, Durance, Vaughan, & Ormerod, 2016).

In freshwater ecosystems, amphipod and isopod crustaceans can be highly abundant, are crucial for the decomposition of organic matter, show an omnivorous feeding pattern and are themselves important food sources for fish and invertebrate predators, thus being important actors in aquatic food webs (Väinölä et al., 2008; Wallace & Webster, 1996). Freshwater amphipods and isopods inhabit a wide range of standing and running water bodies, characterized by various thermal regimes and oxygen conditions. Running waters such as rivers and streams exhibit on average lower temperatures and higher oxygen contents than standing waters such as ponds, reservoirs or stratified lakes (Wetzel, 2001). Hence, species using different habitats are hypothesized to differ in their susceptibility to hypoxia and warming. Similarly, the origin of a species, that is, native or alien, may influence the thermal tolerances under different oxygen conditions. Alien species may have experienced low or changing oxygen conditions during the invasion process, for example, during ballast water transport or crossing different water bodies, explaining why species that successfully spread outside their native range could better cope with hypoxia and heat (Bates et al., 2013; Jewett, Hines, & Ruiz, 2005; Lenz et al., 2011). Finally, it has been suggested that large‐bodied animals may be more prone to oxygen limitation (Chapelle & Peck, 1999; Verberk & Bilton, 2011), explaining why warming may benefit smaller animals (e.g. Daufresne, Lengfellner, & Sommer, 2009). However, size dependency of oxygen limitation has been difficult to demonstrate in arthropods, for example, in aquatic pycnogonids (Woods, Moran, Arango, Mullen, & Shields, 2009) and terrestrial insects (Harrison, Klok, & Waters, 2014).

Understanding if and how oxygen contents affect upper thermal limits will improve our ability to predict the susceptibility of ectotherm species to global warming (Chown, 2012) and improve our predictions of invasion risk associated with alien species (Bates et al., 2013), many of which are crustaceans (Leuven et al., 2009; Van der Velde, Rajagopal, Kelleher, Muskó, & Bij de Vaate, 2000). However, few studies have focussed on patterns of heat tolerance in relation to oxygen limitation in aquatic crustaceans (Verberk, Overgaard, et al., 2016), and available studies have yielded mixed outcomes (Ern et al., 2015; Frederich & Pörtner, 2000). Here, we investigate heat tolerance and the extent to which heat tolerance is modulated by dissolved oxygen content in eight species of freshwater peracarid crustaceans, two preferring running waters, two preferring standing waters and four species occurring in both habitats. Half of the species of each group were native and the other half alien. This experimental setup allows us to test whether differences across species in habitat use, origin or both are associated to differences in heat tolerance and whether patterns in heat tolerance vary with oxygen content. We hypothesize that (1) heat and lack of oxygen synergistically reduce survival of species; (2) oxygen conditions have a stronger influence on the thermal tolerance of native species than alien species and on the thermal tolerance of those that prefer running rather than standing waters; and (3) small animals better tolerate heat compared with large animals and that this difference is more pronounced when oxygen is most limiting (i.e. under hypoxic conditions). We also tested whether differences in thermal tolerance between our study species were related to their oxygen demand (e.g. Verberk & Bilton, 2011), by collating published data on oxygen consumption rates in response to temperature and ambient oxygen conditions.

## 2 MATERIALS AND METHODS

### 2.1 Animal collection and maintenance

Heat tolerance was assessed for four native and four alien species of peracarid Crustacea, which were collected in The Netherlands and Germany. Individuals of the native *Asellus aquaticus* (Linnaeus, 1758) were sampled from a pond in the city of Nijmegen (N51°49′13″, E5°52′28″), *Gammarus fossarum* Koch, in Panzer, 1835 from a brooklet (N51°49′24″, E5°56′32″), *Gammarus pulex* (Linnaeus, 1758) from a ditch (N51°55′18″, E6°40′47″) and *Gammarus roeselii* Gervais, 1835 from a lowland brook (N51°44′17″, E5°57′19″). The alien *Crangonyx pseudogracilis* Bousfield, 1958 was collected from a ditch in the vicinity of Lienden (N51°55′46″, E5°31′41″), individuals of *Dikerogammarus villosus* (Sowinsky, 1894) were collected from the River Waal (N51°51′22″, E5°52′55″), individuals of *Echinogammarus berilloni* (Catta, 1878) from a smaller river near the city of Hamm (North Western Germany (N51°37′55″, E7°55′57), and *Gammarus tigrinus* Sexton, 1939 from the Zijkanaal C of the Noordzeekanaal near Spaarndam (N52°25′13″, E4°41′42″) in March and August 2014. In addition to their origin (native vs. alien), species also differed in their preferred habitat. *A. aquaticus* and *C. pseudogracilis* are more often found in standing waters, whereas *D. villosus* and *G. fossarum* prefer running waters. *E. berilloni, G. pulex, G. roeselii,* and *G. tigrinus* are found in both types of habitat (Eggers & Martens, 2001; Verberk, Verdonschot, Van Haaren, & Van Maanen, 2012; Wijnhoven, Van Riel, & Van der Velde, 2003). Individuals were maintained in the laboratory at 10°C in Dutch standard water (demineralized water with 0.20 g/L CaCl_2_·2H_2_O, 0.18 g/L MgSO_4_·7H_2_O, 0.10 g/L NaHCO_3_, and 0.02 g/L KHCO_3_; pH = 7.9 ± 0.1 and conductivity = 576 ± 44 μS/cm; NEN6503, 1980) at a 12 L:12 D light regime and fed with living chironomid larvae ad libitum. Before recording critical temperatures, all animals were acclimated for at least 7 days to reduce variability in thermal history (Terblanche, Deere, Clusella‐Trullas, Janion, & Chown, 2007). Animals were also gradually acclimated to Dutch Standard Water.

### 2.2 Heat tolerance trials

To assess the heat tolerances of the various species under various oxygen levels, animals were placed in five parallel flow‐through chambers (70 × 70 × 30 mm) using the experimental setup described in detail elsewhere (Verberk & Bilton, 2011). In these flow‐through chambers, we could manipulate water temperature by means of a Grant R5 water bath with a GP200 pump unit (Grant Instrument Ltd, Cambridge, UK) connected to the heat exchanger and oxygen conditions by bubbling nitrogen‐oxygen gas mixtures obtained with a WITT gas‐mixer KM 100‐3 MEM (1) (WITT‐Gasetechnik GmbH & Co KG, Salinger Feld, Germany). Flow rate per chamber was 0.016 L/sec, resulting in a refresh time of 9–10 s. The flow‐through chambers were enriched with artificial stones (made of white modelling clay) providing substrate for individuals to cling on to. After 1 h of acclimatization in the flow‐through chambers with Dutch Standard Water, the water temperature of 10°C was increased at 0.25°C min^−1^ by a water bath with a pump unit (Grant R5, GP200 pump, Grant Instrument Ltd, Cambridge, UK). Temperatures were logged using a HH806AU digital thermometer (Omega Engineering Inc., Stamford, CT, USA). Three different endpoints were noted. During heating, animals increased pleopod movement, until pleopod movement became irregular (first endpoint). Further heating resulted in pleopod movement faltering and the temperature when pleopod movement ceased for more than 2 s was taken as the second endpoint which was on average 1.7°C higher. Finally, all pleopod movement stopped, and the corresponding temperature was defined as the critical maximum temperature (CTmax). This temperature, on average 2.3°C higher, could be most consistently scored and exhibited the lowest variability. Therefore, we focused our analysis on this last endpoint, but note that all three endpoints where strongly related (GLMM using species as a random factor: *F*_1,229_ > 67.71; *p* < .0001; conditional *R* ^2^ > .73). Temperatures were increased for a further 8 min (2°C) after the last individual stopped moving its pleopods to make sure that ceasing of pleopod movement was not transient. The pleopod movements of the exposed individuals were assessed visually. As the pleopods of *A. aquaticus* are ventrally situated, tilted mirrors were inserted in each flow‐through chamber to enable the observation of pleopod movements of this species.

Trials were performed under hypoxia (5 kPa Oxygen), normoxia (20 kPa) and hyperoxia (60 kPa). Temperature and oxygen contents were checked during the experiments via a HH806 AU digital Thermometer (Omega Engineering In., Stamford, CT, USA) and an optical Fibox 3 LCD‐trace oxygen meter (PreSens, Precision Sensing GmbH, Regensburg, Germany), respectively.

At the end of each warming trial, each individual was sexed and its biomass (wet weight after blotting the animals dry to remove adhering water) was determined. For the heat tolerance trials, only non‐gravid individuals were used as the occurrence of eggs may alter the pleopod movement frequency and oxygen requirements. Individuals differed in body mass and individual variation was larger across species than within species: *A. aquaticus* (32.8 ± 2.4 mg wet weight, *M* ± *SE*), *C. pseudogracilis* (8.7 ± 0.2 mg), *D. villosus* (62.9 ± 4.3 mg), *E. berilloni* (22.6 ± 1.6 mg), *G. fossarum* (22.1 ± 1.1 mg), *G. pulex* (50.2 ± 2.3 mg), *G. roeselii* (51.9 ± 2.8 mg) and *G. tigrinus* (11.7 ± 0.7 mg).

### 2.3 Published data on oxygen consumption rates

We collated data on oxygen consumption rates published in the scientific literature for our study species. Studies were included that reported oxygen consumption rates at different temperatures or at different levels of oxygen. We found 25 studies that reported such data for 6 out of 8 species (no oxygen consumption data were retrieved for *C. pseudogracilis* and *E. berilloni*).

To assess whether thermal responses in oxygen consumption rates differed across species, we calculated activation energy (Ea values), which express the thermal sensitivity of oxygen consumption. In most cases, one Ea value was calculated for each species in a given study, but some studies separately reported data for males and females or for different populations and allowed gender‐ or location‐specific calculations of Ea values. In total, 33 Ea values for six species were calculated. Whether animals were acclimated to the test temperature for at least 24 hr prior to measurements of their metabolic rate (acclimated response) or not (acute response) was also noted, as this has been shown to affect the thermal sensitivity (Seebacher, White, & Franklin, 2015).

To assess whether species differed in their respiratory responses when subject to hypoxia, we extracted the critical oxygen level (Pc) from figures and tables, yielding 23 values for four species (*A. aquaticus*,* G. fossarum*,* G. pulex* and *G. roeselii*) and expressed these as a percentage of oxygen levels at normoxia. The critical oxygen level marks the shift from oxygen regulation (whereby metabolic rate is independent of ambient oxygen levels) to oxygen conformation (whereby metabolic rate is largely dependent of ambient oxygen levels). Thus, oxygen regulators can maintain a constant oxygen consumption until the critical oxygen level, whereas oxygen conformers cannot. In practice, this distinction is rarely absolute, and even in oxygen regulators, oxygen consumption rates at the critical oxygen level may have dropped relative to the initial oxygen consumption rates at normoxia (e.g. Brodersen, Pedersen, Walker, & Jensen, 2008; Mueller & Seymour, 2011). Therefore, in addition to the critical oxygen level (Pc), we also noted the oxygen consumption rates at Pc (expressed as a % of those at normoxia). Temperatures employed during these measurements were also noted as respiratory responses when subject to hypoxia are temperature dependent (e.g. Ern, Norin, Gamperl, & Esbaugh, 2016).

### 2.4 Data analysis

To investigate the effects of oxygen content across all species, we constructed a linear mixed‐effects model with species as a random factor (model 1) and a similar model that additionally included body size and the interaction between body mass and oxygen conditions (testing whether potential effects of body mass were dependent on the oxygen conditions) (model 2). Additional models were used to test for an effect of species origin (native or alien) (model 3) and species habitat preference (running waters, indifferent, standing waters) (model 4). The statistical significance of the fixed effects was tested using likelihood ratio tests on models fitted with the function {lme} which were fitted using maximum likelihood. Marginal and conditional pseudo‐*R* ^2^ values were calculated using the function {r.squaredGLMM}, respectively, indicating the variance explained by fixed effects, and by both fixed and random effects, respectively. Contrasts were tested after a Bonferroni correction, using the function {testInteractions}.

To test whether effects of oxygen conditions varied between heat‐tolerant and heat‐sensitive species, we calculated the difference between the mean CTmax under hyperoxia and under hypoxia for each species. Next, we used linear regressions to relate this difference to their mean CTmax observed under normoxia across all eight species. The above calculations were performed in R (version 3.1.1) (R Core Team, 2013), using packages nlme (Pinheiro, Bates, DebRoy, & Sarkar, 2013), mumin (Barton, 2011) and phia (Martinez, 2015).

For each species, differences in CTmax among oxygen treatments were tested using ANOVA with Scheffé post hoc tests when normal distribution and homogeneity of variances were given. In *G. fossarum*, requirements were not met, and Mann–Whitney *U*‐tests with Bonferroni corrections were used. These calculations were performed with PASW (IBM SPSS Statistics, v 22, Chicago, USA).

To test for differences in thermal sensitivity of oxygen consumption (expressed as activation energy, Ea value) between species of different habitat preference or origin, we ran mixed effect models with literature source as a random factor. Preliminary analyses showed Ea values were not significantly influenced by either the thermal range (*p* = .631) over which Ea values were calculated, nor the type of response (acclimated or acute; *p* = .726), so we excluded these factors from the model. Therefore, the model included either habitat preference or origin to test for associated differences in thermal sensitivity of oxygen consumption.

We ran a linear model to test for differences in critical oxygen level (Pc) between species of different habitat preference. We also included the oxygen consumption rate at Pc, expressed as a percentage of the initial oxygen consumption rates at normoxia in our model as preliminary analysis showed that these were related to critical oxygen levels. To prevent overfitting, given the low number of data points (*n* = 23), simpler models were favoured over complex ones. For this reason, we excluded test temperature (*F* = 0.46; *p* = .517) and we did not include literature source as a random factor. Mixed effect models with species as a random factor were not deemed suitable, given that we had three categories of habitat preference and only had data on four species (i.e. 1–2 species for each category). Also, all four species were native of origin, preventing us from testing whether species origin had an effect on the respiratory responses when subject to hypoxia.

## 3 RESULTS

**Figure 1:**
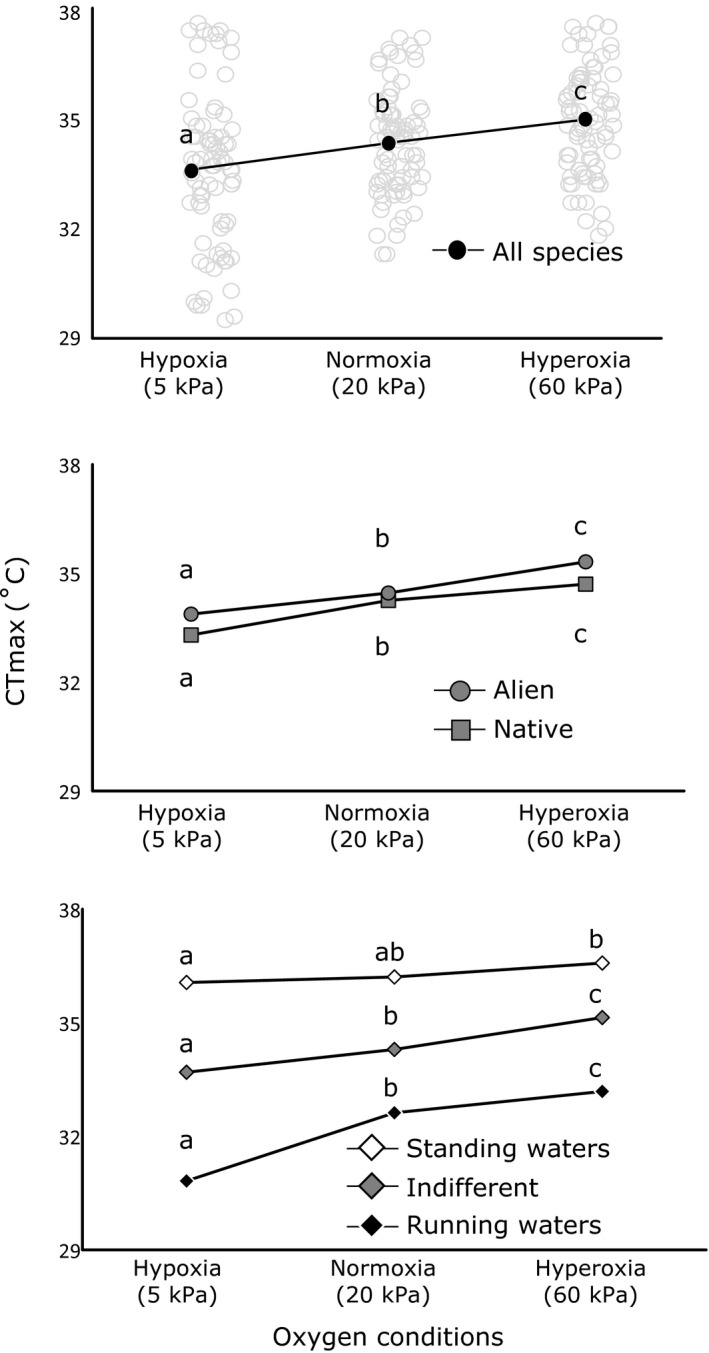
Lethal temperatures (°C) under different oxygen conditions for all species (a), native vs. alien species (b) and species with different habitat use (c). Different letters indicate significant differences between oxygen conditions during heating trials (*p* < .05; as revealed by testing the contrasts of the linear mixed‐effects models with Chi‐square tests and a Bonferroni correction)

Critical thermal maxima varied considerably among treatments and among species, ranging from 30.0 ± 0.9°C (*M* ± *SD*) for *D. villosus* under hypoxic conditions to 37.3 ± 0.4°C for *C. pseudogracilis* under hyperoxic conditions (Table 1; See Figure S1 in Supporting Information). Across all species, oxygen conditions during heating trials significantly affected CTmax (Figure 1; LR test: *p* < .0001), with hypoxia reducing CTmax on average by 0.78°C (*p* < .0001), while hyperoxia significantly improved CTmax by on average 0.66°C (*p* < .0001). When considering species individually, hypoxia significantly reduced CTmax in four species relative to normoxia, while hyperoxia significantly increased CTmax in four species relative to normoxia. When directly comparing hypoxia and hyperoxia, seven species showed an increase in CTmax under hyperoxia (Table 1).

**Table 1:** Lethal temperatures (°C; *M* ± *SD*) of the investigated species for the hypoxia, normoxia and hyperoxia treatment. Different letters indicate significant differences among oxygen treatments (small letters: ANOVA with Scheffé post hoc tests; capital letters: Mann–Whitney *U*‐test with Bonferroni correction)

Species	Hypoxia	Normoxia	Hyperoxia
*Asellus aquaticus*	35.1 ± 0.7 a	35.6 ± 0.5 ab	36.1 ± 0.2 b
*Crangonyx pseudogracilis*	37.3 ± 0.2 a	36.8 ± 0.3 b	37.3 ± 0.4 a
*Dikerogammarus villosus*	30.0 ± 0.9 a	32.3 ± 0.7 b	32.9 ± 0.9 b
*Echinogammarus berilloni*	33.3 ± 0.4 a	34.0 ± 0.6 b	35.0 ± 0.4 c
*Gammarus fossarum*	30.7 ± 0.7 A	32.9 ± 0.4 B	33.4 ± 0.2 C
*Gammarus pulex*	33.8 ± 0.4 a	34.9 ± 0.2 b	35.1 ± 0.4 b
*Gammarus roeselii*	33.1 ± 1.0 a	33.6 ± 0.4 ab	34.2 ± 0.3 b
*Gammarus tigrinus*	34.5 ± 0.5 a	34.7 ± 0.3 a	36.3 ± 0.6 b

Native and alien species did not differ in their CTmax (LR test: *p* = .215) nor in how their CTmax was influenced by oxygen conditions during heating trials (LR test: *p* = .118) (Figure 1b; Table 2). This was also reflected in a higher AIC value for model 3 which included species origin compared to model 2 that did not (502.2 vs. 500.6; Table 2). In contrast, CTmax varied greatly between species from different habitats (LR test: *p* < .0001), with species from standing waters being most heat resistant while those from running waters being most susceptible (Figure 1c; Table 2). Furthermore, the effects of oxygen conditions during heating trials on CTmax were different across species from different habitats (LR test: *p* < .0001). Hypoxia caused the strongest reductions in CTmax in species from running waters (on average 1.8°C), while having negligible effects on species from standing waters (average difference of 0.1°C). Species that were indifferent with respect to water type occupied an intermediate position (average difference of 0.6°C) (Figure 1c). The importance of habitat use as an explanatory factor is also illustrated by the low AIC (438.4) of model 4 and the high marginal *R* ^2^ of .83 (Table 2). Adding body mass and the interaction between body mass and oxygen conditions also improved model fit significantly (*p* < .047 and *p* < .027 for body mass and the interaction, respectively; Table 2). On average, larger animals displayed lower CTmax, and the lower CTmax in larger bodied animals was most prominent under hypoxia (Figure S2).

**Table 2:** Results of the linear mixed‐effects model analyses of the effects of oxygen treatment, species origin (native vs. exotic species) and species habitat use (running water, standing water or indifferent) on the heat tolerance (critical maximum temperature; response variable) of the eight investigated species. Significance of fixed factors was tested by likelihood ratio (LR) tests and the difference in AIC of the model without the fixed factor as compared to the full model is given (negative values indicate that including the fixed factor reduces AIC value [i.e. a better model fit]). Significant fixed factors are indicated boldfaced. For each model, we also provide marginal and conditional pseudo‐*R* ^2^ values (indicating the variance explained by fixed effects, and by both fixed and random effects, respectively) as well as AIC values

Fixed factors	Models			
	1. O_2_	2. O_2_ and body mass	3. As model 2 + origin	4. As model 2 + habitat use
**Oxygen conditions (O**_**2**_ **)**	−139.9139 (*p* < .0001)	−151.715 (*p* < .0001)	−152.0196 (*p* < .0001)	−198.4787 (*p* < .0001)
**Body mass**	–	−11.9126 (*p* = .0005)	−4.7363 (*p* = .0132)	−1.9751 (*p* = .0465)
**O**_**2**_ **× body mass**	–	−13.067 (*p* = .0002)	−5.2567 (*p* = .0098)	−3.24672 (*p* = .0267)
Origin (native or alien)	–	–	1.5302 (*p* = .215)	–
O_2_ × Origin	–	–	−0.2761 (*p* = .1179)	–
**Habitat use**	–	–	–	−62.2585 (*p* < .0001)
**O**_**2**_ **× habitat**	–	–	–	−47.61827 (*p* < .0001)
Model fit				
Marginal *R* ^2^	.10641	.12696	.15559	.83228
Conditional *R* ^2^	.87406	.87518	.87851	.90554
AIC value	512.535	500.623	502.153	438.364

**Figure 2:**
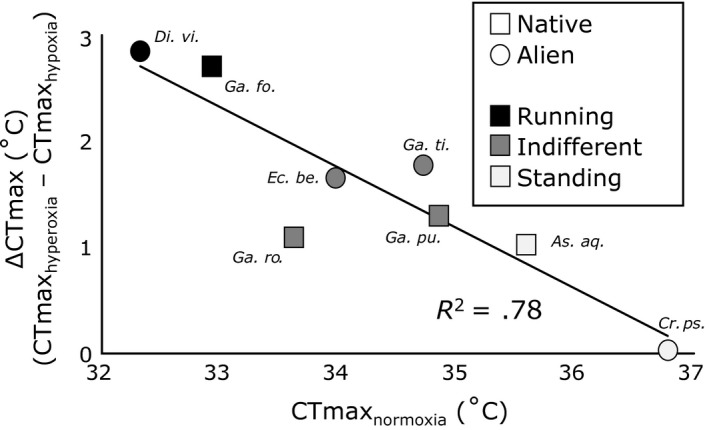
Regression between the difference in lethal temperatures of hyperoxia and hypoxia and the lethal temperatures at normoxia for the investigated species (*As. aq*. = *Asellus aquaticus*,* Cr. ps*. = *Crangonyx pseudogracilis*,* Di. vi*. = *Dikerogammarus villosus*,* Ec. be*. = *Echinogammarus berilloni*,* Ga. fo*. = *Gammarus fossarum*,* Ga. pu*. = *G. pulex*,* Ga. ro*. = *G. roeselii*,* Ga. ti*. = *G. tigrinus*)

The extent to which oxygen conditions affected the outcome of the heating trials, calculated as the difference between CTmax assessed under hyperoxia minus the CTmax when assessed under hypoxia, was clearly different across species. We found a strong negative relationship (adj. *R* ^2^ = .78; *F*_1,6_ = 25.26; *p* = .0024) between the extent to which oxygen conditions modulated CTmax and the average heat resistance when assessed under normoxia (Figure 2). Heat‐tolerant taxa, such as *A. aquaticus* and *C. pseudogracilis*, showed little response to oxygen conditions, whereas the CTmax of heat sensitive species, for example, *D. villosus* and *G. fossarum*, was more plastic, being increased by hyperoxia and strongly reduced by hypoxia.

Thermal sensitivity of metabolic rate, expressed as activation energy (Ea values) calculated from literature data, tended to be slightly higher in animals from standing waters (*t* = 2.81; *p = *.016; Table 3). Ea values did not vary with origin of species (*t* = −0.350; *p* = .732). Reported critical oxygen levels were positively correlated with the ability to maintain oxygen consumption rates at the critical oxygen level such that low critical oxygen levels were frequently accompanied by marked reductions in oxygen consumption rates at these levels (Figure S4). When no critical oxygen levels could be detected (i.e. oxygen consumption rates declined monotonically with falling oxygen levels), critical oxygen levels were taken to equal 100% normoxia where animals by definition fully maintained oxygen consumption rates, which may have strengthened the relationship mentioned above. Differences in respiratory responses when subject to hypoxia across the four species could also be related to habitat preference (Table 3), with the species preferring standing waters (*A. aquaticus*), having relatively low critical oxygen levels (*t* = −4.62; *p* < .001), and in this species, low reported values for critical oxygen levels were not necessarily coupled to marked reductions in oxygen consumption rate at Pc, expressed as a percentage of the initial oxygen consumption rates at normoxia (*t* = 3.633; *p* = .002). No differences in respiratory responses when subject to hypoxia could be detected between the species preferring running waters (*G. fossarum*) and the two species that were indifferent with respect to water type (*G. pulex* and *G. roeselii*).

**Table 3:** Metrics for metabolic rate reported in the literature. Activation Energy (Ea) values (in eV) indicate the thermal sensitivity of oxygen consumption rates. Critical oxygen levels (Pc) are listed alongside with the oxygen consumption rate at Pc, expressed as a percentage of the initial oxygen consumption rates at normoxia (see text for further explanation). Numbers refer to literature sources: 1: Adcock, 1982; 2: Becker, Ortmann, Wetzel, & Koop, 2016; 3: Bruijs, Kelleher, Van der Velde, & Bij de Vaate, 2001; 4: Dorgelo, 1973; 5: Foucreau, Cottin, Piscart, & Hervant, 2014; 6: Franke, 1977; 7: Hervant, Mathieu, & Messana, 1998; 8: Issartel, Hervant, Voituron, Renault, & Vernon, 2005; 9: Lukacsovics, 1958; 10: Maazouzi et al., 2011; 11: Micherdzinski, 1958; 12: Mösslacher & Creuzé des Châtelliers, 1996; 13: Nilsson, 1974; 14: Pieper, 1978; 15: Prus, 1976; 16: Rotvit & Jacobsen, 2013; 17: Roux & Roux, 1967; 18: Roux, 1975; 19: Roux, Roux, & Opdam, 1980; 20: Rumpus & Kennedy, 1974; 21: Suomalainen, 1958; 22: Toman & Dall, 1998; 23: Walshe‐Maetz, 1956; 24: Wautier & Troiani, 1960; 25: Woynárvich, 1961

Species	Ea value ± *SE*	Literature sources	Critical oxygen level (Pc) (% of normoxia)	Metabolic rate at Pc (% of normoxia)	Literature sources
*Asellus aquaticus*	0.87 ± 0.14	1, 15, 16	15.41 ± 6.12	63.22 ± 16.31	7, 12, 16
*Dikerogammarus villosus*	0.54 ± 0.084	2, 3, 9, 10			
*Gammarus fossarum*	0.41 ± 0.11	2, 4, 8, 14, 17, 18	30.00 ± 4.59	63.73 ± 4.59	6, 7, 11, 22
*Gammarus pulex*	0.49 ± 0.062	5, 10, 13, 16, 17, 18, 20, 21, 24	50.40 ± 12.64	74.82 ± 8.95	16, 21, 22, 23, 24
*Gammarus roeselii*	0.38 ± 0.027	2, 9, 19, 25	26.55 ± 26.55	58.73 ± 41.27	22
*Gammarus tigrinus*	0.52	4			

## 4 DISCUSSION

It is important to understand interactive effects of warming and water oxygenation as it is becoming increasingly clear that the effects of each stressor are not merely additive for many aquatic taxa, including molluscs, insects and fish (Koopman, Collas, Van der Velde, & Verberk, 2016; McBryan, Anttila, Healy, & Schulte, 2013; Verberk, Overgaard, et al., 2016). Studies investigating heat tolerance in aquatic crustaceans have mainly focused on heat tolerance under normoxic conditions (Wijnhoven, Van Riel, & Van der Velde, 2003; but see Ern et al., 2015 who also manipulated oxygen conditions). Here, we report patterns in heat tolerance across eight crustacean species in relation to water oxygen content. When averaged across all species, small (<1.0°C) but statistically significant effects were found on heat tolerance, not only for hypoxia but also for hyperoxia, the latter being considered a stronger test of oxygen‐limited thermal tolerance (Verberk, Overgaard, et al., 2016). While these effects were generally small, there is evidence for mayflies that small effects of oxygen on lethal limits become more pronounced for sublethal limits manifested at lower temperatures under field conditions (Verberk, Durance, et al., 2016). Previous work on *A. aquaticus*, one of the most heat‐ and hypoxia‐resistant species in our study, also showed strong interactive effects of temperature and oxygen on growth (Hoefnagel & Verberk, 2015), while employing temperatures (14–24°C) and oxygen levels (10–40 kPa) more moderate than those used in the present study. Still in half of the species we studied, thermal tolerance was not affected by hypoxia or by hyperoxia, reflecting the mixed results reported earlier (Ern et al., 2015; Frederich & Pörtner, 2000). Furthermore, the lowest and highest CTmax recorded did differ by more than 7°C (Table 1) and an explicit aim of this study was, therefore, to investigate if these differences in heat tolerance and their sensitivity to oxygenation can be explained from differences in origin or habitat use of species.

Our hypothesis that alien species would be consistently more heat tolerant was not confirmed. In fact, the alien *D. villosus* was one of the most heat‐sensitive species, confirming the results by Maazouzi, Piscart, Legier, and Hervant (2011) who also observed greater heat tolerance in the native *G. pulex* than in *D. villosus*. However, this pattern contrasts those of other studies on crustaceans (Kenna, Fincham, Dunn, Brown, & Hassall, 2017; Sareyka et al., 2011) and ectotherms more generally (Bates et al., 2013; Lenz et al., 2011; Verbrugge, Schipper, Huijbregts, Van der Velde, & Leuven, 2012). If we investigate more species, this result could change, but at the very least, it suggests that classifying species based on their origin (native or alien) to derive the taxa vulnerability to global warming or to predict invasion success should be done with caution.

Our hypothesis that large‐bodied animals would be more susceptible to heat stress was partly supported by the data. Larger animals displayed lower CTmax under hypoxia, where oxygen limitation is most likely to occur. Size dependency of thermal tolerance implies size dependency of the mechanism setting thermal tolerance. This mechanism has been suggested to be oxygen limitation (Chapelle & Peck, 1999; Pörtner, 2010; Verberk et al., 2011), and limits to oxygen provisioning are implicated in setting upper body size limits (e.g. Kaiser et al., 2007; Lane et al., 2017). However, other studies did not find evidence for size dependency of oxygen limitation (Harrison et al., 2014; Woods et al., 2009). Only when comparing stonefly nymphs spanning several orders of magnitude in size, did Verberk, Sommer, Davidson, and Viant (2013) observe a weak negative relationship between body size and CTmax. In the present study, body mass also explained only a small amount of the variation in heat tolerance under hypoxia (*c*.1°C difference across a 10‐fold difference in body mass).

Most of the variation in heat tolerance across species was linked to differences in their habitat use (Figure 1c), with model four showing the highest marginal *R* ^2^ of .83 (Table 2). Moreover, the extent to which oxygen‐limited heat tolerance differed across species differing in habitat use. In a recent review on arthropods, Verberk, Overgaard, et al. (2016) suggested that whether taxa show oxygen limitation at thermal extremes may be contingent on their capacity to regulate oxygen uptake, which in turn, could help explain differences in support for oxygen limitation between aquatic and terrestrial ectotherms. Indeed, studies on insects and freshwater snails indicate that the extent to which taxa show oxygen‐limited thermal tolerance depends on the ability of animals to regulate oxygen uptake (Boardman & Terblanche, 2015; Koopman et al., 2016; Verberk & Bilton, 2013, 2015). All the species investigated in our study have similar gas exchange mechanisms (i.e. aquatic gas exchange across respiratory surfaces such as the gills and coxal plates). However, species in standing waters are more frequently confronted with periods of low oxygen while conditions for oxygen uptake should be consistently better in running waters where permanent water flow greatly reduces the ventilation effort needed, while the resultant mixing of air and water ensures high water oxygenation. The adaptations that species possess to cope with such environmental conditions in their preferred habitat likely also help to explain differences in heat tolerance and their sensitivity to oxygenation. Adaptations to ensure adequate oxygen provisioning under heat or hypoxia may explain why oxygen had little effect on the heat tolerance of the two most tolerant species (*A. aquaticus* and *C. pseudogracilis*), and these may involve low oxygen demand or high capacity for oxygen uptake (via enhanced ventilation or circulation). *A. aquaticus* was shown to be an oxygen conformer (Table 3; Rotvit & Jacobsen, 2013), which may explain its five times higher tolerance to hypoxia compared to *G. pulex* (Maltby, 1995). While no data on respiratory responses when subject to hypoxia could be retrieved for *C. pseudogracilis*, it was shown to be five times more tolerant to hypoxia compared with *G. pulex* (Macneil & Dick, 2014). Sareyka et al. (2011) compared two amphipod species and reported greater hypoxia tolerance in the most heat‐tolerant species. In addition, to respiratory responses when subject to hypoxia, differences between species in their thermal sensitivity of oxygen demand, expressed as Ea values, may explain their thermal tolerance. Stonefly nymphs with high Q_10_ values have been reported to have a lower CTmax (Verberk & Bilton, 2011), which seems straightforward when high Q_10_ values indicate that the oxygen demand of an individual is very sensitive to increasing temperatures, making it more susceptible to oxygen‐limited heat tolerance. Yet, our analysis on Ea values extracted from the literature showed higher Ea values in animals preferring standing waters if anything (Table 3). The problem here is that a high Ea value may be taken as a proxy for increases in oxygen demand with temperature, but in order to satisfy such increases in demand, an animal needs sufficient capacity to extract and transport oxygen. Consequently, without data on scope for oxygen consumption across temperatures, high Ea values could also be interpreted as a high capacity for oxygen delivery under warmer temperatures. Both viewpoints may be correct but in different contexts, such that oxygen consumption transitions from being demand driven to being supply driven when metabolic rates approach maxima (see also Verberk, Bartolini, et al., 2016).

Intriguingly, our results show that the inherent heat tolerance (CTmax under normoxia) is a strong predictor for the extent to which oxygen content affects heat tolerance (the difference between CTmax when assessed under hypoxia and hyperoxia) (Figure 2). This suggests that oxygen limitation could be involved in setting thermal limits in heat‐susceptible taxa, but that in heat‐resistant taxa, the role of oxygen is smaller (*A. aquaticus*) or even absent (*C. pseudogracilis*) and mechanisms other than insufficient oxygen become more influential. The relatively low number of species in our study precluded us from taking phylogeny into account. Other studies did not found a clear association between thermal tolerance and phylogeny across amphipods and isopods (Best & Stachowicz, 2013). In our case, the two species preferring standing water are both distantly related to the remaining species, and they are also distantly related to each other (Figure S5). The most divergent species *A. aquaticus*, a descendent of the Asellota already occurring in the Triassic (Wilson, 2009), did not exhibit the most divergent physiology, which was exhibited by *C. pseudogracilis*. Similarly, *G. pulex* and *G. fossarum* which are very closely related (Hou & Sket, 2016; Figure S5) showed quite divergent physiology, and differences between these species were consistent with the pattern of oxygen having smaller effects in heat‐resistant taxa. Thus, the relationship reported in Figure 2 does not seem to be confounded by phylogenetic relationships, but studies encompassing a greater number of species allowing phylogenetic relatedness to be taken into account are needed to verify this intriguing observation.

Our study has implications for both predicting susceptibility to global warming and invasion success. Species from running waters (e.g. *G. fossarum* and *D. villosus*) are likely to be more susceptible to interactive effects of warming and hypoxia than those of standing waters (e.g. *A. aquaticus* and *C. pseudogracilis*), but at the same time, running water taxa may profit most from improvements in water quality (e.g. reduction in nutrient load), and such improvements may even off‐set the effects of warming (Durance & Ormerod, 2009; Verberk, Durance, et al., 2016). It is striking that *D. villosus* appears most susceptible to hypoxia and heat stress. This may explain why this species is very successful in colonizing new areas, but mainly in the rocky substrate with continuous wave action, flow and water displacements by passing ships or prevailing winds, resulting in continuous aeration (Gabel, 2012; Gabel et al., 2008; Platvoet, Dick, MacNeil, Van Riel, & Van der Velde, 2009). This likely also explains why this species is much less adept in spreading to other waters which are less well oxygenated and where *C. pseudogracilis* is the more likely alien species.

In conclusion, oxygen availability had stronger effects in crustaceans that prefer running waters, and these taxa were also found to be more heat susceptible. Conversely, taxa that prefer standing waters were more heat resistant and had better oxyregulatory capacity, and heat tolerance was much less affected by oxygen availability. Such context‐dependence effects of oxygen on thermal limits could potentially reconcile the mixed support for the oxygen and capacity limitation of thermal tolerance hypothesis (Ern et al., 2015; Frederich & Pörtner, 2000).

## DATA ACCESSIBILITY

Data available from the Dryad Digital Repository https://doi.org/10.5061/dryad.tf641 (Verberk, Leuven, van der Velde, & Gabel, 2018).
