# Supplementary material for: Thermal limits in native and alien freshwater peracarid Crustacea: The role of habitat use and oxygen limitation
Source: Funct Ecol. 2018 Feb 6;32(4):926–36. doi: 10.1111/1365-2435.13050 (PMC5993316; doi:10.1111/1365-2435.13050)
Supplement: Supplementary file 1 [file FEC-32-926-s001.pdf]

Functional Ecology

BRITISH ECOLOGICAL SOCIETY

Habitat use explains vulnerability to hypoxia and global warming in aquatic invertebrates

Wilco C.E.P. Verberk, Rob S.E.W. Leuven, Gerard Van Der Velde, Friederike Gabel

Climate warming has sparked interest in the heat tolerance of animals and the ecophysiology underpinning their thermal tolerance. One idea suggests that ectotherms or cold blooded animals succumb to heat because they run out of oxygen. This is because warming increases their energy requirements which in turn pushes up their oxygen demand. Understanding the underlying ecophysiology could be useful for conservation, but also for predicting invasion success of alien or non-native species that invade ecosystems beyond their historic range. In this study we examine 8 species of aquatic crustaceans of which 4 were native and 4 were non-native to the Netherlands. We measured how well they could tolerate heat and whether their heat tolerance was influenced by water oxygenation. We found that they succumbed at temperatures between 30 and 37 degrees, indicating a considerable range in sensitivity to heat. This variation in heat tolerance could be partly explained by differences in water oxygenation. Heat tolerance tended to be lower under poor water oxygenation compared to normal or above normal water oxygenation. Additionally, species differed in their heat sensitivity. Non-native species were not more heat tolerant than native species. Instead, the preferred habitat of the species proved decisive: species preferring running waters had lower heat tolerance than those from standing waters, reflecting the fact that running waters tend to be cooler. The successful invader *D. villosus* appeared most susceptible to hypoxia and heat stress. This may explain why this species invades predominantly shores with rocky substrate which are well aerated due to continuous wave action generated by passing ships. Interestingly, the influence of water oxygenation was found to be greater in heat sensitive species preferring running waters. This suggests that the ecophysiological mechanisms underlying heat tolerance change from heat sensitive to heat tolerant taxa. This indicates that improving water oxygenation (by reducing organic pollution or increasing flow) can help to offset the effects of global warming, especially in running waters.

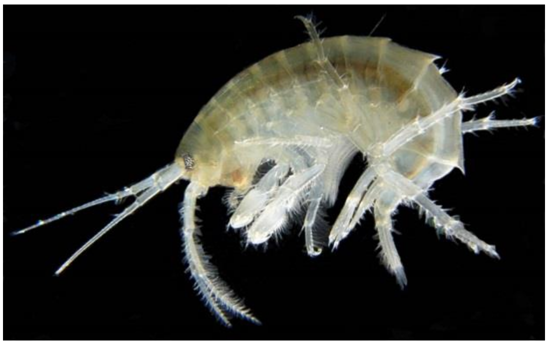

Gammarid amphipods are key players in the decomposition of leaf litter. Depicted is Gammarus roeselii, one of the species studied. Photo credit: Michal Maňas
