# Supplementary material for: Thermal limits in native and alien freshwater peracarid Crustacea: The role of habitat use and oxygen limitation
Source: Funct Ecol. 2018 Feb 6;32(4):926–36. doi: 10.1111/1365-2435.13050 (PMC5993316; doi:10.1111/1365-2435.13050)
Supplement: Supplementary file 2 [file FEC-32-926-s002.docx]

**Figure S1**. Lethal temperatures (°C) under the different oxygen conditions for eight peracarid Crustacea.

**Figure S2.** Body mass plotted against residual variation in CTmax (taking into account differences among species by showing the residuals of a mixed effect model that only included species as a random factor), showing that larger animals have reduced CTmax under hypoxia (red), but less so under hyperoxia (black) or normoxia (green) (Likelihood ratio-test, oxygen x body mass interaction: P< 0.0267; see Table 2).

**Figure S3**. Boxplot of the thermal sensitivity of oxygen consumption (expressed by Ea values) extracted from the literature (See Table 3).

**Figure S4**: Scatterplot of two components of respiratory responses when subject to hypoxia: the critical oxygen level and the oxygen consumption rate at this level (both expressed as a % of normoxia). Oxygen regulators can maintain high oxygen consumption rates down to low oxygen levels (upper left part of the figure), whereas oxygen conformers have high critical oxygen levels at which their oxygen consumption rates are strongly reduced (lower right part of the figure).

**Figure S5.** Simplified scheme of phylogenetic relationships among our eight study species (based on Hou & Sket (2016) and Wilson (2009). Branch lengths are not drawn to scale; Asellota already occurring in the Triassic (>200 Mya ago), whereas the main radiation of the Gammaridae occurred during the Paleogene (approx. 38 Mya ago). Also, several groups of Crustacea, such as the Tanaidacea and Cumacea, are not included in this phylogeny for clarity, but are located in between the isopods and amphipods.
